# Editorial Notes

**Published:** 1883-02

**Authors:** 


### EDITORIAL NOTES.

THE remarkable endorsement which *Dyspepsin* has received from the profession proves conclusively the good quality and value of the preparation. We are informed by druggists that the sale of it is constantly increasing.

THE new year has brought with it several changes in our contemporaries. The *New York Medical Journal*, the *Chicago Medical Review* and *The Sanitarian* have become weeklies; while several of the weeklies, notably the *New York Medical Record* and *The Louisville Medical News*, have been increased in size. All of the journals mentioned are ably edited and deserve the prosperity of which we trust these changes are an indication.

THE constant increase in the circulation of the JOURNAL affords the editors no little pleasure as an evidence that their labors are not in vain. The demand for back numbers also has so far exceeded our expectations that we are now unable to supply certain numbers. Any of our subscribers who do not preserve their volumes will do us a favor by mailing to us the numbers for April, July, August and September, 1882. We will credit their account with the full price of JOURNALS returned, 25 cents for each number.

WE desire to call attention to some valuable medical preparations which are worthy of receiving greater attention from medical men. Messrs. Wyeth & Bro. are furnishing compressed tablets of Bisulphate of Quinine, which are readily soluble and cannot fail to dissolve when swallowed. While the writer was in Philadelphia, he critically examined the method followed by Wyeth & Bro. in preparing their compressed pills and other pharmaceuticals, with the result of decidedly increasing his previous high opinion of their preparations.

